# Risk of early failure of VP shunts implanted for hydrocephalus after craniotomies for brain tumors in adults

**DOI:** 10.1007/s10143-021-01549-7

**Published:** 2021-04-27

**Authors:** Sayied Abdol Mohieb Hosainey, John K. Hald, Torstein R. Meling

**Affiliations:** 1 Department of Neurosurgery, Bristol Royal Hospital for Children, Bristol, UK; 2 Department of Radiology and Nuclear Medicine, Oslo University Hospital, Oslo, Norway; 3 Faculty of Medicine, Institute of Clinical Medicine, University of Oslo, Oslo, Norway; 4 Department of Neurosurgery, Oslo University Hospital, Oslo, Norway; 5 Department of Neurosurgery, Geneva University Hospitals, Geneva, Switzerland; 6 Faculty of Medicine, University of Geneva, Geneva, Switzerland

**Keywords:** Neurosurgery, Brain tumor, Hydrocephalus, VP shunt, Survival, Complications

## Abstract

Risks and survival times of ventriculoperitoneal (VP) shunts implanted due to hydrocephalus after craniotomies for brain tumors are largely unknown. The purpose of this study was to determine the overall timing of VP shunting and its failure after craniotomy for brain tumors in adults. The authors also wished to explore risk factors for early VP shunt failure (within 90 days). A population-based consecutive patient cohort of all craniotomies for intracranial tumors leading to VP shunt dependency in adults (> 18 years) from 2004 to 2013 was studied. Patients with pre-existing VP shunts prior to craniotomy were excluded. The survival time of VP shunts, i.e., the shunt longevity, was calculated from the day of shunt insertion post-craniotomy for a brain tumor until the day of shunt revision requiring replacement or removal of the shunt system. Out of 4774 craniotomies, 85 patients became VP shunt-dependent (1.8% of craniotomies). Median time from craniotomy to VP shunting was 1.9 months. Patients with hydrocephalus prior to tumor resection (*N* = 39) had significantly shorter time to shunt insertion than those without (*N* = 46) (*p* < 0.001), but there was no significant difference with respect to early shunt failure. Median time from shunt insertion to shunt failure was 20 days (range 1–35). At 90 days, 17 patients (20%) had confirmed shunt failure. Patient age, sex, tumor location, primary/secondary craniotomy, extra-axial/intra-axial tumor, ventricular entry, post-craniotomy bleeding, and infection did not show statistical significance. The risk of early shunt failure (within 90 days) of shunts after craniotomies for brain tumors was 20%. This study can serve as benchmark for future studies.

## Introduction

Craniotomies for removal of brain tumors form the core treatment of these potentially deadly diseases and have been proven to prolong life [31, 39] and improve quality of life and overall survival [20, 33]. Nonetheless, infections [24, 26, 27], bleeding [13, 27], surgical morbidity/mortality including neurological sequelae [2, 27], and CSF disturbances [16–18, 26] are potential risks of surgery.

Hydrocephalus has been extensively studied with abundant evidence for its treatment with procedures such as external ventricular drainage (EVD), endoscopic third ventriculostomy (ETV), and ventriculoperitoneal (VP) shunts [14, 16, 21, 30]. Although the main objective of treating hydrocephalus with VP shunts is to establish a permanent CSF diversion, achieving maximum VP shunt survival, defined as time from implantation to its malfunction, still remains challenging. Numerous studies have been published on postoperative shunting and shunt-survival rates with respect to the pediatric population [16], hemorrhage-related hydrocephalus [28, 34], infections [4, 8, 24, 25], shunting related to specific tumor types [3, 19], and vascular brain malformations [15]. However, studies on shunt-survival rates and risks leading to shunt failure with respect to brain tumors remain scarce.

In this study, we wished to determine timing of VP shunting and its longevity, and also to determine the risk of early shunt failure (within 90 days) implanted due to hydrocephalus after craniotomies for brain tumors in adults.

## Materials and methods

### Collection of data

A population-based consecutive patient cohort of all adult patients operated at a single regional health care center between 2004 and 2013 was reviewed using our prospective database. The following data were recorded: age at time of craniotomy for brain tumor, VP shunt surgery and at time of first shunt failure, sex, status of hydrocephalus before and after craniotomy (yes/no), tumor location (supratentorial/infratentorial), extra-axial or intra-axial tumor (based on tumor histology and imaging reported by neuroradiologists), primary/secondary (repeated) tumor resection, histology, treatment modality for hydrocephalus before and after craniotomy (EVD/ETV/EVD and tumor surgery simultaneously), ventricular entry during craniotomy (yes/no), post-craniotomy bleeding (yes/no), and post-craniotomy infection/meningitis (yes/no). The first/index craniotomy in a specific location was defined as primary craniotomy and all subsequent craniotomies in the same location were defined as secondary. Therefore, a patient could have had more than one primary craniotomy, if operated on multiple/different locations. Secondary/repeated craniotomy for brain tumor was also designated for those who had craniotomies before the study period (2004), but later craniotomies within the study period. No patients were lost to follow-up.

To identify patients who underwent EVD, ETV, and VP shunting before and/or after brain tumor surgery, our tumor database was cross-linked with our surgical procedure codes database using the Nordic Medico-Statistical Committee Classification of Surgical Procedures (NCSP) codes for CSF-related procedures (operation codes AAF). ICD-10 codes (G91) were subsequently reviewed to verify each case. Biopsy cases were not included in this study. Patients with pre-existing VP shunts prior to their craniotomies were excluded from the study.

The time from craniotomy for brain tumor to shunt insertion and the time from shunt insertion to shunt failure were recorded. VP shunt failure was suspected based on clinical signs and symptoms of altered intracranial pressure and radiological signs of ventricular enlargement, such as prominent temporal horns, increased transverse diameter of third ventricle > 5 mm, ballooning of frontal horn with periventricular changes on CT, or T2-weighted/FLAIR images. For patients with shunt dependency, we recorded whether there was any significant association between ventricular entry during craniotomy and shunt longevity. We also recorded post-craniotomy bleeding (intraparenchymal and/or intraventricular hemorrhage) and infection (positive CSF and device cultures including CSF pleiocytosis with clinical picture of infection requiring shunt removal) for analysis of shunt longevity. All patients underwent either MRI or CT head imaging at time of suspected shunt failure. Shunt failure was defined as a shunt revision procedure resulting in replacement of the whole shunt or in part by its individual components such as catheter replacement as a result of blockage and/or change or replacement of shunt valve.

The survival time of VP shunts, i.e., the shunt longevity, was calculated from the day of shunt insertion post-craniotomy until the day of shunt revision requiring replacement or removal of the shunt system. Analysis of risk factors associated with early shunt failure was performed to within 90 days after shunt insertion. Censoring at the 90th day post-shunting was chosen in order to determine whether the shunt failure was associated with the brain tumor surgery rather than adjuvant therapies. In order to avoid having multiple counts of the same patients in our analyses and to account for multiple procedures in the same patient, patient-to-craniotomy ratio was ensured to be 1:1 in the final analyses for shunt longevity and its associated risks by excluding duplicate patient identification numbers (IDs). Hence, a patient could have multiple craniotomies, but all patient IDs were unique in the final analyses.

### Statistical analysis

Kaplan–Meier method was used to construct survival curves for shunt-free period, i.e., time from craniotomy to shunt insertion and from first day of VP shunt insertion to date of first revision. For detecting shunt failure with respect to the Kaplan–Meier analysis method, log rank test was applied to determine statistical significance of different risk factors for shunt failure. Cox proportional hazard regression models were used to identify multiple potential predictor variables with respect to time to shunt insertion and to shunt failure. Chi-square (*X*^2^) and Fisher’s exact test were used for comparison between categorical variables. Analysis of variance (ANOVA) and Student’s *t* test were used for continuous variables. Statistical significance was set at *p* < 0.05 and for all analyses, the statistical software JMP (version 9.03) was used.

## Results

### Demographic data

**Fig. 1:**
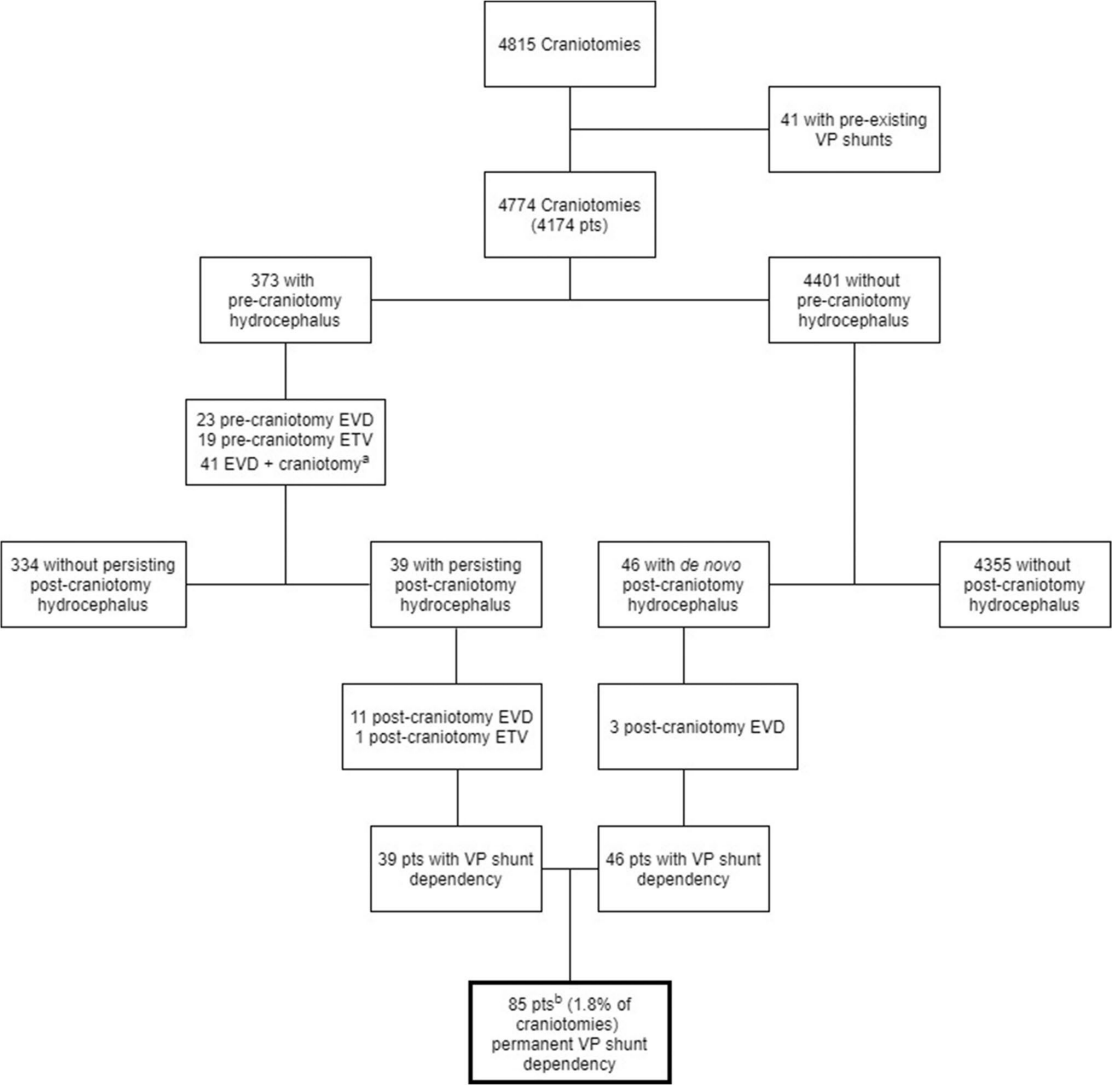
Flowchart illustrating all cases leading to VP shunt dependency after craniotomy for intracranial tumors. VP shunt – ventriculoperitoneal shunt. ^a^: EVD insertion and craniotomy for brain tumor simultaneously. ^b^: 2.0% of patients

A total of 4815 craniotomies for brain tumors were performed on 4204 adult patients. After exclusion of patients with pre-existing VP shunts (30 patients), a total of 4774 cases underwent further analyses (Fig. 1).

Three hundred seventy-three patients had pre-craniotomy hydrocephalus, of which 39 patients had persisting hydrocephalus and became shunt-dependent. Four thousand four hundred and one craniotomy cases had no pre-craniotomy hydrocephalus, but 46 had de novo hydrocephalus and became shunt-dependent (Fig. 1). Thus, out of the 4774 craniotomies, a total of 85 patients (2% of patients, 1.8% of craniotomies) became shunt-dependent (Fig. 1). There were 44 males (48.2%) and 41 females (51.8%) (Table 1). Sixty-eight patients (80.0%) had supratentorial tumors, while 17 patients (20.0%) had an infratentorial tumor location (Table 1). The median patient age at time of shunt insertion was 61.9 years (range 23.5–81.6 years) (Table 2).

**Table 1:** Overview characteristics of patients who underwent craniotomy for brain tumor and required permanent CSF diversion

	Persisting hydrocephalus and VP shunt dependency (*N*/%)	De novo post-craniotomy hydrocephalus and VP shunt dependency (*N*/%)	Total VP shunt dependency after craniotomy (*N*/%)
Total	39	46	85
Age (median yrs)	54.8	62.0	61.9
Sex			
Male Female	19 (48.8) 20 (51.2)	25 (54.3) 21 (45.7)	44 (51.8) 41 (48.2)
Tumor location			
Supratentorial Infratentorial	24 (61.5) 15 (38.5)	44 (95.6) 2 (4.4)	68 (80.0) 17 (20.0)
Extra-axial tumor Intra-axial tumor	17 (43.6) 22 (56.4)	16 (34.8) 30 (65.2)	33 (38.8) 52 (61.2)
Surgery			
Primary Secondary	30 (77.0) 9 (23.0)	34 (73.9) 12 (26.1)	64 (75.3) 21 (24.7)
Histology			
HGG Meningioma Metastasis Other tumors Ependymoma Craniopharyngioma Schwannoma Choroid plexus tumor Pituitary adenoma LGG	10 (25.6) 9 (23.0) 5 (12.8) 4 (10.3) 4 (10.3) 2 (5.1) 1 (2.6) 1 (2.6) 1 (2.6) 2 (5.1)	11 (23.9) 12 (26.1) 13 (28.3) 4 (8.7) 0 2 (4.3) 2 (4.3) 1 (2.2) 1 (2.2) 0	21 (24.7) 21 (24.7) 18 (21.2) 8 (9.5) 4 (4.7) 4 (4.7) 3 (3.6) 2 (2.3) 2 (2.3) 2 (2.3)
Pre-craniotomy and post-craniotomy treatment for hydrocephalus			
Pre-craniotomy EVD Pre-craniotomy ETV EVD + craniotomy simult Post-craniotomy EVD Post-craniotomy ETV	2 (5.1) 2 (5.1) 7 (17.9) 11 (28.2) 1 (2.6)	0 0 0 3 (6.5) 0	2 (2.3) 2 (2.3) 7 (8.2) 14 (16.5) 1 (1.2)
Ventricular entry during craniotomy	2 (5.1)	7 (15.2)	9 (10.6)
Post-craniotomy bleeding	4 (10.3)	4 (8.7)	8 (9.4)
Post-craniotomy infection	2 (5.1)	2 (4.3)	4 (4.7)

*EVD* external ventricular drainage; *ETV* endoscopic third ventriculostomy; *VP* ventriculoperitoneal

**Table 2:** Time frame of different variables with respect to shunting after craniotomy for brain tumor

	Days from craniotomy to VP shunting (range)^a^	Days from VP shunting to failure—shunt longevity (range)^a^	VP shunt failure within 90 days (*N*/%)
Age (median)	61.0 years (range 33.3–74.6)	61.1 years (range 33.4–74.6)	61.1 years (range 33.4–74.6)
Sex			
Male Female	87 (13–194) 48.5 (17–376)	20 (1–35) 19.5 (3–24)	9 (52.9) 8 (47.1)
HC prior to craniotomy			
Yes^b^ No^c^	16 (13–22) 87 (26–376)	4.5 (1–24) 20 (2–35)	4 (23.5) 13 (76.5)
Tumor location			
Supratentorial Infratentorial	50.5 (13–376) 56 (-)	19.5 (1–35) 21 (-)	16 (94.2) 1 (5.8)
Intra-axial Extra-axial	40 (24–92) 41 (26–66)	20 (1–30) 19 (2–23)	9 (52.9) 8 (47.1)
Surgery			
Primary Secondary	56 (13–376) 67 (15–194)	20 (1–24) 18 (3–35)	13 (76.5) 4 (23.5)

*HC* hydrocephalus, *VP* ventriculoperitoneal

^a^Times given as median unless otherwise specified

^b^Cases with persisting postoperative HC (after craniotomy) requiring VP shunting

^c^Cases with de novo (new onset) postoperative HC requiring VP shunting

### Time to shunt insertion after craniotomy for brain tumor

**Fig. 2:**
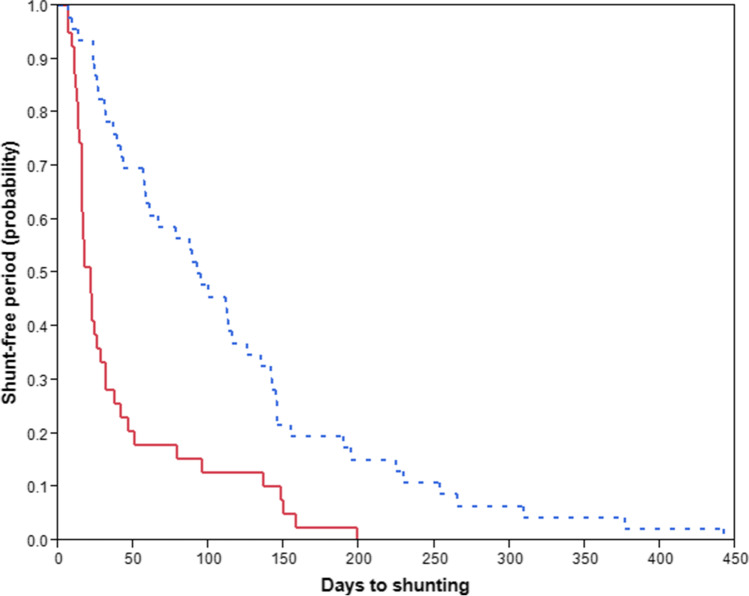
Kaplan–Meier curves demonstrating the overall time from craniotomy to shunt insertion (shunt-free period). Red continuous and blue-dotted curves represent those with and without hydrocephalus prior to craniotomy, respectively

The median time from craniotomy to VP shunt insertion was 1.9 months (range 0.4–12.5 months) (Table 2). Patients with hydrocephalus before craniotomy had a significantly higher risk of earlier VP shunt dependency than those without preoperative hydrocephalus (Fig. 2) in both univariate (HR 2.7, CI [1.7–4.3], *p* < 0.001) and multivariate (HR 3.7, CI [2.1–6.5], *p* < 0.001) analyses (Table 3).

**Table 3:** Survival and risk analysis of shunting with univariate and multivariate proportional hazards ratio model

	Time to VP shunting (risk of VP shunt implantation)		Shunt longevity (risk of early failure from shunting)	
	Univariate (HR, CI [95%])	Multivariate (HR, CI [95%])	Univariate (HR, CI [95%])	Multivariate (HR, CI [95%])
Age (median)				
Craniotomy for brain tumor Shunt insertion Shunt failure	1.0 [1.0–1.1] 1.0 [1.0–1.1] N/A	1.0 [1.0–1.1] 1.0 [1.0–1.1] N/A	1.0 [0.9–1.1] 1.0 [0.9–1.1] 1.0 [0.9–1.1]	1.0 [0.9–1.1] 1.0 [0.9–1.1] 1.0 [0.9–1.1]
Sex				
Male Female	1 1.0 [0.6–1.5]	1 0.9 [0.6–1.6]	1 0.8 [0.4–1.5]	1 0.9 [0.2–3.9]
Pre-craniotomy hydrocephalus				
No Yes	1 2.7^a^ [1.7–4.3]	1 3.7^a^ [2.1–6.5]	1 1.2 [0.5–5.7]	1 2.0 [0.3–11.1]
Tumor location				
Supratentorial Infratentorial	1 0.9 [0.5–1.6]	1 1.3 [0.6–2.6]	1 0.6 [0.4–1.1]	1 1.1 [0.1–9.8]
Extra-axial tumor Intra-axial tumor	1 0.9 [0.6–1.5]	1 1.1 [0.6–1.9]	1 0.7 [0.2–1.9]	1 0.9 [0.2–3.2]
Surgery				
Primary Secondary	1 0.7 [0.4–1.1]	1 0.6 [0.3–1.1]	1 1.7 [0.9–3.2]	1 0.1 [0.1–2.2]
Hydrocephalus treatment modality perioperatively (craniotomy)				
Pre-craniotomy EVD Pre-craniotomy ETV EVD + craniotomy simultaneously Post-craniotomy EVD Post-craniotomy ETV	4.1 [0.7–12.3] 4.7 [0.7–16.5] 1.7 [0.7–3.6] 1.1 [0.5–1.8] 1.3 [0.1–6.2]	–^b^ 2.4 [0.3–11.7] –^b^ 2.1 [0.9–4.9] 1.8 [0.4–29.8]	–^b^ –^b^ 0.9 [0.3–4.1] 5.8 [0.8–29.4] –^b^	–^b^ –^b^ 1.0 [0.1–83.1] 1.0 [0.3–71.2] –^b^
Post-craniotomy bleeding	1.1 [0.5–2.4]	2.2 [0.8–5.6]	1.2 [0.5–2.4]	–^b^
Ventricular entry (craniotomy)	0.9 [0.4–1.7]	1.4 [0.6–2.9]	0.7 [0.1–3.7]	1.4 [0.1–27.3]
Post-craniotomy infection	1.8 [0.5–4.4]	2.4 [0.6–7.1]	1.2 [0.2–4.5]	6.2 [0.6–48.8]

*EVD* external ventricular drain, *ETV* endoscopic third ventriculostomy, *HR* hazard ratio, *CI* confidence interval, *VP* ventriculoperitoneal

^a^*p* < .001

^b^Variable parameters insufficient to determine HR for shunt longevity as there were too few patients who had early shunt failure to reveal statistical significance

Patient age, sex, tumor location, primary/secondary craniotomy, extra-axial/intra-axial tumor, hydrocephalus treatment modality before/after craniotomy, ventricular entry during craniotomy, post-craniotomy bleeding, and post-craniotomy infection did not show statistical significance in neither univariate nor multivariate analysis (Table 3).

### Risk of shunt failure

At time of censoring (90 days), 17 patients (20.0%) had undergone revision procedures with confirmed shunt failure (median 20 days, range 1–35) (Table 2). There were 4 patients with and 13 patients without hydrocephalus before craniotomy with median shunt longevity of 4.5 and 20 days, respectively. Median age at time of shunt failure was 61.1 years. There was no statistically significant association between those with and without hydrocephalus prior to craniotomy and reduced shunt longevity within 90 days (Table 3).

### Age and sex

Median age at time of shunt failure was 61.1 years (range 33.4–74.6). Age at craniotomy, VP shunting, and at time of shunt failure was not significantly associated with shunt longevity neither in univariate nor in multivariate analysis (Table 3).

Of the 85 patients who became shunt-dependent, 44 patients (51.8%) were male and 41 patients (48.2%) were female (Table 1). Sex was not significantly associated with overall shunt longevity neither in univariate nor in multivariate proportional hazards analyses (Table 3).

### Tumor location

Sixty-eight patients (80.0%) had supratentorial tumor location at time of craniotomy for brain tumor, while 17 patients (20.0%) had infratentorial tumor location (Table 1). Tumor location was not significantly associated with shunt longevity neither in univariate nor in multivariate proportional hazards analyses (Table 3).

### Extra-axial and Intra-axial tumors

There were a total of 33 out of 85 patients (38.8%) whom had extra-axial tumors of which 17 patients (43.6%) had pre-craniotomy hydrocephalus, while the remaining 52 patients (61.2%) had intra-axial tumors (Table 1). No statistical significant differences were detected between those with extra-axial/intra-axial tumors and time to neither VP shunting (*p* = 0.9) nor shunt longevity (*p* = 0.5) in neither univariate nor multivariate analysis (Table 3). Stratified risk analysis with respect to pre-craniotomy status of hydrocephalus revealed no statistical significant difference between these two patient groups in time to VP shunting (with pre-craniotomy hydrocephalus: HR 0.8, CI [0.4–1.5], *p* = 0.4; without pre-craniotomy hydrocephalus: HR 1.1, CI [0.6–1.9], *p* = 0.8) and early shunt failure within 90 days (with pre-craniotomy hydrocephalus: HR 2.1, CI [0.9–1.1], *p* = 0.06; without pre-craniotomy hydrocephalus: HR 2.2, CI [0.6–7.8], *p* = 0.2).

### Primary/secondary surgery for brain tumor

Primary craniotomies were performed in 64 patients (75.3%) and secondary in 21 patients (24.7%) (Table 1). Primary/secondary craniotomy for brain tumor was not significantly associated with shunt longevity neither in univariate nor in multivariate proportional hazards analyses (Table 3).

### Pre-craniotomy and post-craniotomy treatment for hydrocephalus

Out of the 373 patients with pre-craniotomy hydrocephalus, 23 underwent EVD insertion, 19 had ETV, while 41 had EVD and craniotomy concomitantly (Fig. 1). From these, 334 patients (89.5%) did not need any further treatment for hydrocephalus. Of the remaining 39 patients with persisting post-craniotomy hydrocephalus, 11 had EVD insertion and one underwent ETV in an attempt to avoid shunting. Ultimately, all 39 patients with persisting post-craniotomy hydrocephalus required VP shunt insertion (Table 1). From the 39 patients with VP shunt dependency, two had pre-craniotomy EVD and two had ETV, while seven patients had EVD and tumor resection simultaneously (Table 1). Except one patient, there was no overlap of treatment modalities for hydrocephalus in the pre-craniotomy and post-craniotomy stage before VP shunt implantation (Fig. 1).

From those without pre-craniotomy hydrocephalus, 3 out 46 patients had EVD insertion after tumor surgery due to de novo hydrocephalus, but all 46 patients required VP shunt insertion eventually (Table 1).

Of the total of 85 patients with shunt dependency, none had craniotomies between initial shunt placement and first shunt failure. Having a pre-craniotomy treatment for hydrocephalus was not significantly associated with reduced shunt longevity (Table 3).

### Histology

From a total of 85 patients with shunt dependency, the tumor histologies were as follows in descending order: 21 high-grade gliomas (24.7%), 21 meningiomas (24.7%), 18 metastatic tumors (21.2%), 8 other tumors (9.5%), 4 ependymomas (4.7%), 4 craniopharyngiomas (4.7%), 3 schwannomas (3.6%), 2 choroid plexus tumors (2.3%), 2 pituitary adenomas (2.3%), and 2 low-grade gliomas (2.3%) (Table 1).

### Ventricular entry during craniotomy

Nine out of 85 patients (10.6%) with shunt dependency had ventricular entry during craniotomy (Table 1). Only one patient (1.2%) had early shunt failure. Ventricular entry was not significantly associated with reduced shunt longevity (Table 3).

### Post-craniotomy bleeding

Eight out of 85 patients (9.4%) with shunt dependency had post-craniotomy bleeding (Table 1). None of these patients had early shunt failure. Post-craniotomy bleeding was not significantly associated with reduced shunt longevity (Table 3).

### Infection

Only 4 out of 85 patients (4.7%) with shunt dependency had infection after craniotomy and initial shunt surgery (Table 1). From these, 2 patients (2.3%) had early shunt failure. Infection was not significantly associated with reduced shunt longevity (Table 3).

## Discussion

Craniotomies for brain tumors carry a risk of causing postoperative hydrocephalus in need of VP shunts or ETVs for permanent CSF diversion [10, 21]. However, VP shunts may malfunction and studies on shunt longevity and potential risks leading to early shunt failures after brain tumor surgery remain unexplored. We have previously reported the incidence and risk factors of developing postoperative hydrocephalus in patients with and without hydrocephalus before brain tumor surgery [17, 18]. Therefore, the primary end-point of this study was to investigate differences with respect to time to VP shunting, in particular between these two groups (patients with and without pre-craniotomy hydrocephalus). Also, we wished to analyze shunt longevity within the first 90 days after shunting. Our secondary end-point was to determine risk factors of early VP shunt failure that can lead to reduced shunt longevity after craniotomy for brain tumors.

A total of 85 patients (1.8% of craniotomy cases, 2% of patients) became permanently shunt-dependent after craniotomies for brain tumor (Fig. 1, Table 1). The median time to VP shunt insertion after craniotomy was 1.9 months (Fig. 2, Table 2). In comparison, studies have reported time to shunt placement after surgeries ranging from the day of initial biopsy to 15 months postoperatively in adults with high-grade gliomas [3, 11, 19, 37]. In a study by Reddy et al. on shunting of patients with intracranial tumors, 56 out of 187 patients (30%) had a shunt placement after tumor surgery [36], but the time to shunt placement after craniotomy was not stated. While the abovementioned studies are limited to particular tumor types and patient populations, our study included all adult patients comprising a non-selected consecutive cohort with histologically verified intracranial tumors, thus strengthening the external validity of our study.

Adult patients without pre-craniotomy hydrocephalus who have undergone craniotomy for choroid plexus tumors and craniopharyngiomas have been shown to have higher risk of post-craniotomy shunt dependency [18]. This has also been demonstrated for adults with pre-craniotomy hydrocephalus and who have undergone secondary surgery [17]. In this study, comparative analysis of these two states of with/without preoperative hydrocephalus at time of tumor surgery was performed. This revealed that hydrocephalus prior to craniotomy was significantly associated with shorter time between craniotomy and VP shunting compared to those without hydrocephalus before craniotomy regardless of preceding treatment for hydrocephalus with EVD or ETV prior to definite shunting (Fig. 2, Table 3). A possible explanation for this might be that the tumor burden causing obstruction of the CSF pathways and overloading venous outflow leads to considerable alterations in the overall CSF dynamics of the brain [38, 40]. The “stasis” of CSF and the effect of the pathophysiologic state may cause cell death and axonal damage [5, 6], changes in brain elasticity [22], and profound tissue edema in addition to ventriculomegaly, which in turn prolongs the hydrocephalic state intracranially even after tumor resection. As such, those with persistently abnormal intracranial pressures and ventriculomegaly post-resection will have earlier post-craniotomy hydrocephalus due to persisting hydrocephalus and subsequent shunt dependency, an effect which is stronger than in those without any changes in CSF dynamics apart from the local peritumoral edema within the brain parenchyma with no disturbance of CSF pathways. Comparable to our previous findings [17, 18], age at craniotomy and shunt placement, sex, tumor location, and primary/secondary surgery were not significant predictors of time to shunt insertion in this study (Table 3).

**Fig. 3:**
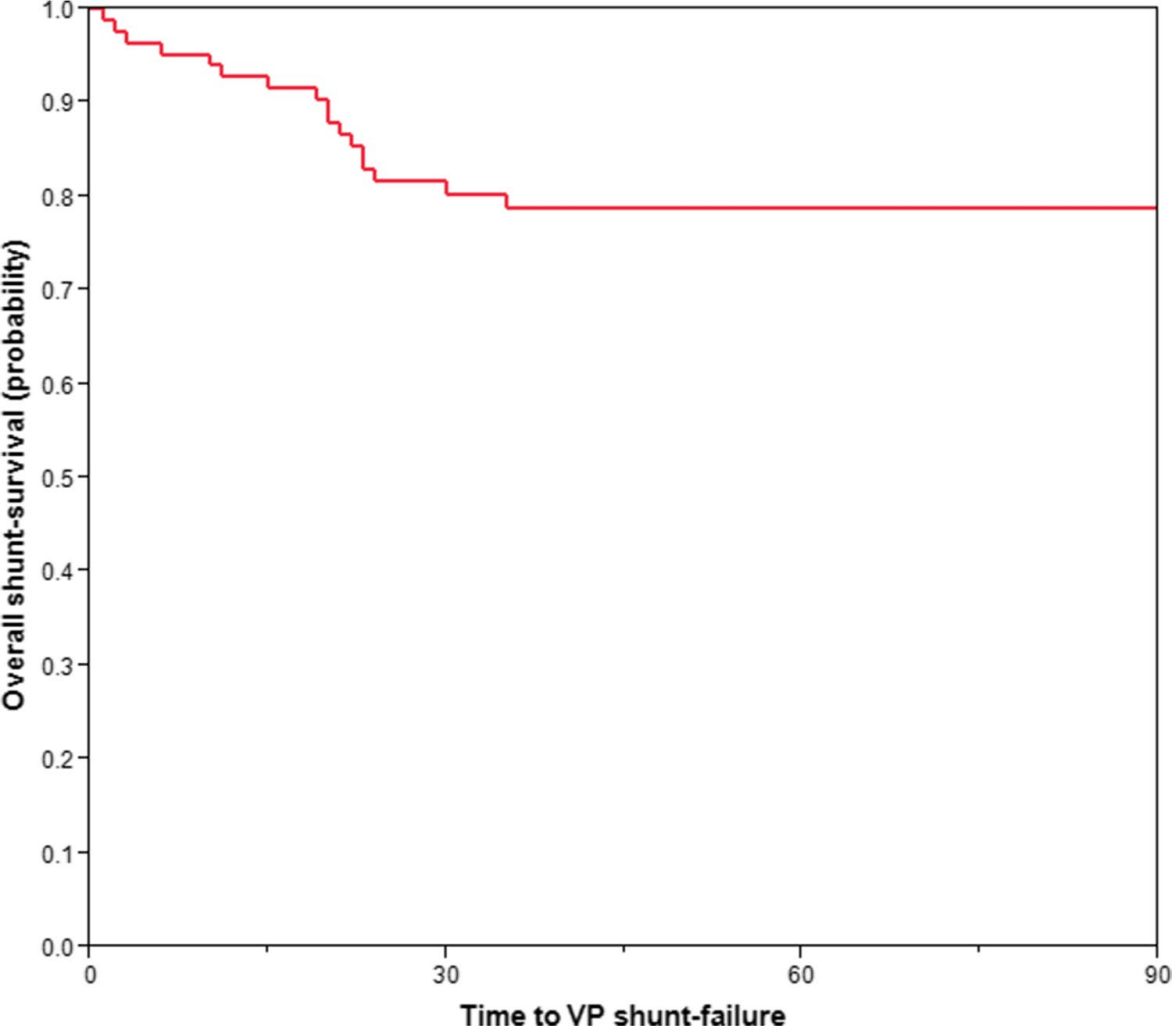
Kaplan–Meier curve demonstrating overall VP shunt survival (within 90 days) after brain tumor surgery

At time of censoring (90 days), 17 patients (20%) had confirmed shunt failure with median time of 20 days and median age at first shunt failure was 61.1 years (Table 2), yielding a 90-day shunt-survival rate of 80% after craniotomy for brain tumor (Fig. 3). Notably, only 4 of these 17 patients had hydrocephalus before craniotomy, while the remaining 13 patients presented without hydrocephalus before craniotomy for brain tumor (Table 2). In the literature, shunt-failure rates range from 16.9 to 28.8% at 3 months [4, 7, 36] and 9.17–77.3% at 6 months [7, 8, 23–25, 36] with median shunt-survival times from 22.5 days up to 5.2 years [23, 25, 35]. However, most of these studies have not been conducted of brain tumor patients as a separate cohort. We found that neither patient age nor hydrocephalus existing prior to craniotomy was a significant risk factor for early shunt failure (Table 3). Some studies have identified younger adults as risk for shunt failure [7, 24], whereas others have not [8]. In a study by Anderson et al. [1] of etiologies of shunt failures in adults, the hydrocephalus etiology (idiopathic, infection, or trauma) was found to be a significant risk factor for 30-day shunt failure, whereas age was not. Reddy et al. reported a 2% decrease in odds for shunt failure with increasing age at shunt placement [36]. Interestingly, we found that patients with hydrocephalus before craniotomy had significantly higher risk of earlier shunting, but were not more likely to have longer shunt longevity compared to those without hydrocephalus prior to brain tumor surgery (Figs. 2 and 3, Table 3). This might be explained by brain tumor debris within the CSF after brain tumor surgery leading to earlier hydrocephalus and subsequent shunt dependency. Additionally, one could expect that patients with earlier VP shunting would have reduced shunt longevity, even though the discrepancy of time to shunting and shunt failure between those with and without prior hydrocephalus was comparable (Table 2). Although high protein content from certain tumor types has been associated with hydrocephalus [11, 32], other factors such as hemoventriculi at the time of shunt surgery [28], male sex, and benign tumors have been associated with shorter shunt survival [36]. Lastly, Reddy et al. [36] reported that females had significantly longer shunt-survival rates (*p* < 0.001) compared to males; we could not find this association in our study.

With respect to treatment modalities for hydrocephalus before and after tumor surgery, EVDs were inserted in 66 out of 373 cases (17.7%) with pre-craniotomy hydrocephalus and in 2 out of 4401 cases (0.1%) without pre-craniotomy hydrocephalus (Fig. 1). In total, 334 out of 373 cases (89.5%) with pre-craniotomy hydrocephalus and 2 out of 4401 cases (0.1%) did not require permanent shunting post-craniotomy. EVD placement (both independent of and concomitant with craniotomy) or ETVs was not significantly associated with time to neither VP shunting nor shunt longevity (Table 3). In a study by Won et al., 71% of patients received preoperative and perioperative EVD placements, whereas one patient received EVD postoperatively due to hydrocephalus. Preoperative hydrocephalus was a significant risk factor for development of postoperative hydrocephalus [41], similar to our findings but risk of EVD for shunt failure was not analyzed in their cohort of patients limited to posterior fossa lesions. All patients in their study underwent VP shunting (mean interval from surgery in adults was 69.7 ± 109.9 days). Korinek et al. reported that previous EVD placements and male sex were risk factors for first revision for mechanical shunt dysfunction. The cause of hydrocephalus had no impact on risk of shunt dysfunction [25]. Conversely, Lee et al. reported that EVD placement at index surgery (defined as new shunt or revision for the patient) was not predictive of 30-day shunt failure [28]. However, both of the abovementioned studies were not limited to brain tumor patients only. Furthermore, their studies have not given detailed specifics with regard to the underlying conditions for requirement of EVD placements. With regard to ETV for pre-craniotomy hydrocephalus treatment, none had persisting post-craniotomy (*N* = 19) hydrocephalus leading to shunt dependency in our study. One patient had pre-craniotomy ETV and subsequent insertion of EVD which successfully avoided shunting. Other studies have reported ETV success rates ranging from 73 to 98% with miscellaneous etiologies of hydrocephalus [14, 21, 30]. Marx et al. reported in their study of adults with posterior fossa lesions only that there was no significant difference in developing persisting hydrocephalus (with subsequent shunting) between those who had tumor surgery only and those with additional perioperative ETV [30], in keeping with our results. Reddy et al. reported that patients who had procedures such as ventriculostomy and Ommaya reservoir had significantly lower 3 and 6-month shunt-survival rates than those without these procedures [36]. As only 3 out of 39 patients in total (7.7%) had ETV from those with pre-craniotomy and post-craniotomy hydrocephalus, our statistical analyses were non-interpretable with respect to shunt longevity. Nonetheless, our higher ETV success rate for hydrocephalus treatment may be explained by inclusion of adults with brain tumors only.

There were 33 patients (38.8%) out of 85 patients whom had extra-axial tumors in this study. Extra-axial tumors such as choroid plexus tumors, craniopharyngiomas [18], schwannomas [12], and ependymomas [23] are known to increase the risk of postoperative hydrocephalus. This might lead to requirement of permanent CSF diversion. However, when dichotomizing patients into extra-axial and intra-axial tumors, our analyses did not reveal any statistical significance between these two groups in time to VP shunting nor early shunt failure (Table 3). Also, we did not find any statistically significant difference when further stratifying our analyses into patients with/without pre-craniotomy hydrocephalus. This might partially be explained by some extra-axial tumor types being truly intraventricular ones, whereas others are not located in the vicinity of the ventricles and do not require ventricular opening during resection, which might increase the risk of postoperative hydrocephalus development [11, 32]. Nonetheless, our study comprises a limited number of patients where contemporary studies are lacking with regard to extra-axial and intra-axial tumors and postoperative VP shunting and early shunt failure in this patient group.

In our study, secondary/repeat surgery was not associated with increased risk of early shunt failure within 90 days. The median time to shunt failure was similar between those who underwent primary and secondary craniotomy (18 days vs. 20 days, respectively). Secondary/repeat surgery is associated with postoperative hydrocephalus in patients with pre-craniotomy hydrocephalus [17] and in development of communicating hydrocephalus in patients with glioblastomas [32], contrary to the pediatric population where no statistical significance has been found [16]. Although we could not associate secondary/repeat craniotomy for brain tumor with shunt longevity, further studies are warranted as comparison to our study was difficult due to lack of reports in the literature.

High-grade gliomas and meningiomas represented approximately half of the cases with shunt dependency (Table 1). Postoperative hydrocephalus leading to permanent shunt dependency has been reported with regard to both malignant [11, 19, 32] and benign tumors [18]. Moreover, malignant brain tumors have been reported to have significantly lower shunt revision rates compared to benign tumors [36], but no difference compared to control groups (normal pressure hydrocephalus) [37]. In a study of adult patients with brain tumors and development of hydrocephalus, craniotomy for choroid plexus tumors and craniopharyngiomas had higher risk of shunt dependency than other tumor types [17]. In the same study, ventricular entry was not significantly associated with shunt dependency reflecting our current findings in this study, but also with regard to shunt longevity (Table 3). We also did not find any significant relation with post-craniotomy hemorrhage and shunt longevity (Table 3), in contrast to other reports where shunt malfunctions were significantly lower in patients with intracranial hemorrhages [7, 29]. Nonetheless, Lee et al. reported that intraventricular hemorrhage at index surgery was a significant risk factor for shunt failure, but this was at time of new shunt insertion or first revision and not craniotomy. Our results might be explained by that none of the patients with post-craniotomy hemorrhage had ventricular entry during craniotomy for brain tumor (Table 1).

In our study, only 4 patients in total (4.7%) had post-craniotomy infection (Table 1). This is in the lower end of the scale compared to published reports [1, 24, 25, 36]. Korinek et al. also identified that previous EVD placements and previous craniotomy have increased risk of shunt revision due to shunt infection [25]. Ferguson et al. reported that post-meningitic hydrocephalus patients (three patients) had significantly longer shunt-survival time (28 months) than other causes [9]. We did not find any significant association between post-craniotomy infection and time to shunting or shunt longevity (Table 3). Our lower total incidence rate of post-craniotomy infection may be explained by inclusion of only adults who underwent surgery for brain tumors.

### Strength and limitations of the study

The centralized neurosurgical health care center at Oslo University Hospital (Rikshospitalet and Ullevål) has a population-based referral of patients from a well-defined geographical region of Norway with approximately 2.8 million inhabitants. This centralization of neurosurgical services reduces possible confounding effects of differences in access to health care services. We have avoided the selection bias inherently present in large multicenter studies, as there is only one unit performing neurosurgical procedures. Our study is unique in that we did not find any other large-scale studies with focus on analysis of shunt survival and possible risks associated with shunt failure after craniotomies for brain tumors where all patients are included regardless of tumor histology. This study comprises histological specters which are clinically relevant, thereby improving the external validity of our results. There is no selection bias, as all consecutive craniotomies from a prospectively collected database with histologically verifiable intracranial tumors are included. Finally, no patients have been lost to follow-up and to the extent of our knowledge, this is the largest study with respect to analyzing shunt survival and risks associated with shunt failure in patients who become shunt-dependent after craniotomy for brain tumors.

The foremost limitation of this study is its retrospective design. Potential selection bias might be evident because of surgeon’s preferences for treatment with EVD and/or ETV as well as timing for shunting due to hydrocephalus. Factors such as tumor volume, shunt valve type, and details of the mechanical components of the shunt devices such as calcification within the tube leading to scarring, exact site of shunt blockage, and malpositioning/migration of catheter were not included in our analyses. The analysis of images with regard to shunt failure was not performed in an automatized manner, due to lack of comparability across the different imaging modalities in absence of age-adjusted normal values. Although CT/MRI was available for all patients included in the study, the presence or absence of ventriculomegaly leading to shunt failure and subsequent revision may have been limited by human error. Adjuvant treatments such as radiotherapy, chemotherapy, and coexisting comorbidities were not included in the analyses, which may impact the risks associated with shunt failures. Although being a large study, the number of patients might be so low in the final analyses giving statistical type I and II errors, thus failing to identify true prognostic factors for shunt failures. Direct comparisons to our study were difficult as most published reports are biased with limitations to certain patient groups, tumor histologies and accounting for overall shunt-failure rates, whereas our study was concerned to adults whom had brain tumor surgery.

## Conclusions

A total of 1.8% of cases with intracranial tumors had permanent shunt dependency. Median time from craniotomy for brain tumor to VP shunting was 1.9 months. In total, 89.5% of those with pre-craniotomy hydrocephalus did not require a shunt postoperatively. Hydrocephalus prior to craniotomy for brain tumor was significantly associated with earlier shunt insertion, but not with early shunt failure within 90 days. In adult patients who underwent craniotomy for brain tumor, only 20% had shunt failure within 90 days.

## Data Availability

Data may be given upon reasonable request.
